# Diosmetin Exerts Synergistic Effects in Combination with 5-Fluorouracil in Colorectal Cancer Cells

**DOI:** 10.3390/biomedicines10030531

**Published:** 2022-02-24

**Authors:** Sareh Kamran, Ajantha Sinniah, Zamri Chik, Mohammed Abdullah Alshawsh

**Affiliations:** Department of Pharmacology, Faculty of Medicine, University of Malaya, Kuala Lumpur 50603, Malaysia; sarehkamran.sk@gmail.com (S.K.); ajantha.sinniah@um.edu.my (A.S.); zamrichik@ummc.edu.my (Z.C.)

**Keywords:** synergistic effect, dose reduction index, combination index, diosmetin, 5-fluorouracil, colorectal cancer

## Abstract

5-Fluorouracil (5-FU) is a chemotherapeutic medication commonly used to treat colorectal cancer (CRC); however, the drug-associated adverse effects and toxicity have greatly affected its clinical use. Exploring another therapeutic strategy that lowers the toxicity of 5-FU while having a synergistic effect against CRC is thus a viable option. Diosmetin, a natural flavonoid, has been shown to inhibit the proliferation of many cancer cells, including CRC cells. This study aims to investigate the synergistic effect of diosmetin and 5-FU on HCT116 and HT29 colorectal cancer cells and to explore the apoptotic activity of this combination. The MTT assay was used to assess the viability of cells treated with monotherapy and combination therapy. The combination index (CI) and dose reduction index (DRI) were calculated using the CompuSyn software (version 1.0). The SynergyFinder 2.0 software was used to calculate the synergy score, while the Combenefit software was employed to perform isobologram analysis and synergism determination. The AO/PI double staining technique was used to detect the apoptotic characteristics of cells, whereas the flow cytometry technique was used to investigate the apoptosis induction and cell cycle arrest in cells. The combination of 5-FU and diosmetin showed a synergistic effect in HCT116 cells with a mean CI value of 0.66 ± 0.4, and an additive effect in HT29 cells with a CI value of 1.0 ± 0.2. The DRI of 5-FU in HCT116 cells was three times lower in the combination therapy compared to monotherapy of 5-FU. AO/PI microscopic examination and Annexin V analysis revealed that the combination-treated cells had more apoptotic cells than the monotherapy-treated cells, which was activated mainly through intrinsic apoptosis pathway. HCT116 cell death was confirmed by mitotic arrest in the G2/M phase. Our findings suggest that 5-FU/diosmetin combination exhibits synergistic effect against HCT116 cancer cells, and potentially reduces the unfavorable adverse effect of 5-FU while enhancing the anticancer efficacy by inducing apoptosis and interrupting mitosis. Further research studies are needed to validate the combination’s anti-tumorigenic activities in a xenograft animal model.

## 1. Introduction

**Figure 1:**
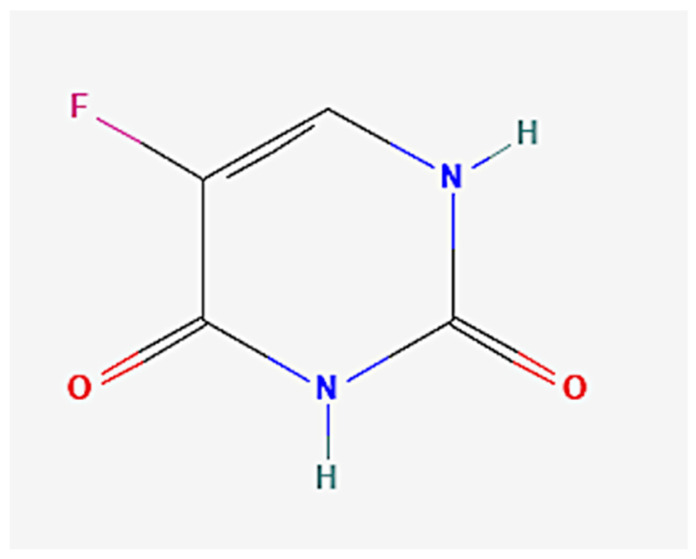
Chemical structure of 5-FU. https://pubchem.ncbi.nlm.nih.gov/compound/5-Fluorouracil (accessed on 28 January 2022).

Colorectal cancer (CRC) is the third most prevalent cancer worldwide and the second most deadly cancer. In 2020, GLOBOCAN estimated 1,148,515 new CRC cases and 576,858 colorectal cancer deaths [1]. Although there have been substantial advances in the chemotherapeutic agents against CRC, severe adverse effects and toxicity are the major clinical problems. Hence, exploring other potential therapeutic strategies to combat CRC is crucial [2]. The synergistic effect of existing chemotherapeutic drugs in combination with natural and safe bioactive agents is an effective approach that has been considered in previous studies [3,4,5,6,7]. There are several chemotherapeutic drugs such as doxorubicin, cisplatin, methotrexate, 5-fluorouracil (5-FU), and paclitaxel that are used in combination therapy [8]. The current combination therapies include FOLFOX regimen (folinic acid + 5-FU + oxaliplatin), FOLFIRI regimen (folinic acid + 5-FU + irinotecan), XELOX or CAPOX regimen (capecitabine and oxaliplatin), and CAPIRI regimen (capecitabine + irinotecan) [9]. Although the numbers of new chemotherapeutic agents have increased, usage of 5-FU combined therapy remains a preferred control measurement for the treatment of CRC. However, severe cytotoxic effects and other toxicities are still the main concern for conventional chemotherapy combinations [10]. 5-Fluorouracil (Figure 1) is used as a first-line therapy against CRC and acts by inhibiting thymidylate synthase, causing DNA damage [10]. Due to its high cytotoxicity to normal tissues, a few studies have reported that 5-FU in combination with certain bioactive phytoconstituents may reduce the toxic adverse effects of 5-FU and increase its efficacy [8,11].

**Figure 2:**
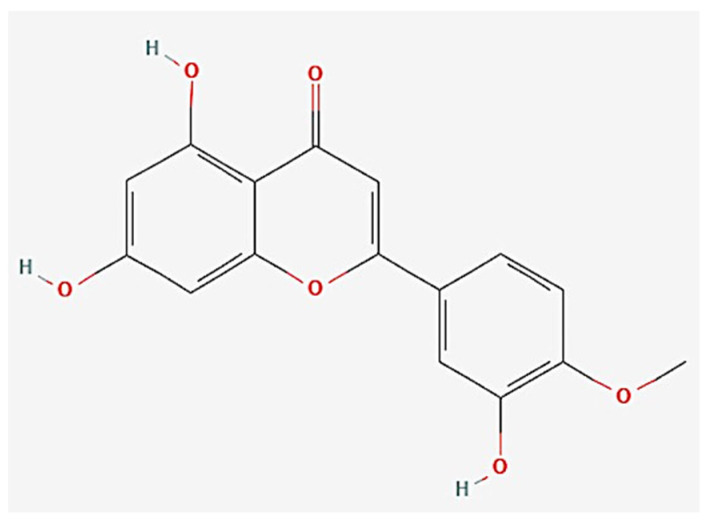
Chemical structure of diosmetin. https://pubchem.ncbi.nlm.nih.gov/compound/Diosmetin#section=Structures (accessed on 28 January 2022).

Diosmetin (Figure 2), a natural flavonoid, is found in a variety of plants such as *Caucasian vetch* and citrus. Some studies have shown that diosmetin confines tumor progression and exerts antiproliferative activities against breast cancer, hepatocarcinoma, lung cancer, prostate cancer, leukemia, and CRC [3,4,5,6,12]. The molecular mechanisms underlying the anti-cancer activity of diosmetin include cellular arrest at the G2/M phase, activation of apoptosis via Fas and Bax at gene and protein levels, releases of cytochrome C, caspases cascade cleavage, and suppression of NF-_K_B translocation [13]. Moreover, diosmetin has been demonstrated to be more selective toward cancer cells with negligible cytotoxicity toward normal cells, although it is less effective than 5-FU [13]. In this study, 5-FU in lower doses was combined with diosmetin to investigate the synergistic effect of both agents against HCT116 and HT29 colorectal cancer cells and to determine the dose reduction index of 5-FU, as well as to elucidate the apoptotic activities of this combination.

## 2. Materials and Methods

### 2.1. Cell Lines and Culture Condition

HCT116 and HT29 colon cancer cell lines were obtained from American Type Culture Collection (ATCC, Manassas, VA, USA) and were cultured in DMEM media (Capricorn Scientific Gmbh, Ebsdorfergrund, Germany) containing 10% fetal bovine serum (FBS) (Capricorn Scientific Gmbh, Ebsdorfergrund, Germany) and 1% penicillin/streptomycin (Gibco, Billings, MT, USA). The cells were incubated at 37 °C with 5% CO_2_ and 95% humidity.

### 2.2. MTT Assay to Determine Cell Viability

The MTT experiment was conducted to assess the anti-proliferative effect of diosmetin (ab142292, Abcam, Cambridge, United Kingdom) and 5-FU (MP Biomedicals, CA, USA) in a monotherapy model and a combination therapy model in HCT116 and HT29 cells [14]. HCT116 and HT29 cells were seeded at a density of 5 × 10^3^ cell/well and then exposed to a two-fold serial dilution of either 5-FU ranging from 100–0.78 μg/mL (768.8–6.0 μM) or diosmetin ranging from 100–0.78 μg/mL (333.0–2.6 μM). Then, cells were incubated for 72 h. Formazan crystals were dissolved with DMSO and the absorbance was measured at 570 nm using a microplate reader [14]. After the IC_50_ determination, cells were treated with a combination of diosmetin and 5-FU at a constant ratio (1:5) in triplicates. The doses involved in combination therapy were IC_50_, and other doses higher and lower than the IC_50_ of monotherapy [15]. The IC_50_ of combination therapy was determined to calculate the combination index (CI) and dose reduction index (DRI).

### 2.3. Synergistic Effect Analysis

The Chou Talalay equation [15] and CompuSyn software (version 1.0; ComboSyn, Paramus, NJ, USA) were used to determine the CI and DRI. The CI was used to determine the types of drug interactions where CI < 1 indicates synergistic effect, CI = 1 indicates additive effect, and CI > 1 represents antagonistic effect. The equation below was used to calculate the CI for the combination.

(1) $$\mathrm{CI}=\frac{{\mathrm{IC}}_{50}\;\mathrm{of}\;\mathrm{drug}\;5\mathrm{FU}\;\mathrm{in}\;\mathrm{combination}\;}{{\mathrm{IC}}_{50}\;\mathrm{of}\;5\mathrm{FU}\;\mathrm{in}\;\mathrm{monotherapy}}+\frac{{\mathrm{IC}}_{50}\;\mathrm{of}\;\mathrm{diosmetin}\;\mathrm{in}\;\mathrm{combination}\;}{{\mathrm{IC}}_{50}\;\mathrm{of}\;\mathrm{diosmetin}\;\mathrm{in}\;\mathrm{monotherapy}}$$

The dose reduction index (DRI) was calculated using the formula below, measuring how many folds the dose of 5-FU could be reduced in the combination, compared with the monotherapy.

(2) $$\mathrm{DRI}=\frac{{\mathrm{IC}}_{50}\;\mathrm{of}\;5\mathrm{FU}\;\mathrm{in}\;\mathrm{monotherapy}}{{\mathrm{IC}}_{50}\;\mathrm{of}\;5\mathrm{FU}\;\mathrm{in}\;\mathrm{combination}\;}$$

Combenefit software was used to perform isobologram analysis and synergism determination, whereas SynergyFinder 2.0 software was used to determine a single synergy score.

### 2.4. AO/PI Double Staining Assay

Acridine orange (Santa Cruz Biotechnology, CA, USA) and propidium iodide (Santa Cruz Biotechnology, CA, USA) (AO/PI) fluorescent dyes were used to test the microscopic morphology and changes of HCT116 cells following treatment with 5-FU, diosmetin, and a combination of both. Cells were treated for 72 h with the IC_50_ of monotherapy and combination therapy. Then, 10 μL of AO/PI mixture was used to stain the cell pellet. The morphological alterations in the cells were detected using a fluorescent inverted microscope. The resulting green, orange, and red colors represent viable, late apoptotic, and dead cells, respectively [13].

### 2.5. Annexin V/PI Assay for the Detection of Cell Apoptosis

The purpose of this experiment was to determine the apoptotic activities of combination therapy in comparison to monotherapy. Briefly, HCT116 cells were treated for 72 h with IC_50_ of monotherapy and combination therapy. To detect apoptosis, cell pellets were labeled with PI (Santa Cruz Biotechnology, CA, USA) and FITC-Annexin V (BD Biosciences, San Jose, CA, USA) for 15 min before being introduced to the FACS Caliber flow cytometer [13]. 

### 2.6. Cell Cycle Assessment

This assay was conducted to detect demolition in the phases involved in the cell cycle after cellular exposure to monotherapy and combination therapy. Briefly, 2 × 10^6^ HCT116 cells were seeded in a T75 cm^2^ flask followed by overnight incubation. Treatment was conducted via exposing the cells to the IC_50_ of monotherapy and combination therapy for 24, 48 and 72 h. Negative control cells were maintained under the same conditions. After that, trypsinized cells were collected and stabilized via 70% ethanol at −20 °C overnight. After rinsing the cells, they were stained and incubated with 500 μL PI (Santa Cruz Biotechnology, CA, USA) for 30 min at room temperature. Cell cycle assessment was measured using BD FACSCanto II flow cytometer [13]. ModFit LT 5.0 software was used to analyze the cell cycle data.

### 2.7. Caspases Activity Detection

The effect of monotherapy and combination therapy on HCT116 cells was assessed using Caspase-Glo 8, 9, and 3/7 kits (Promega, Wisconsin, USA). Briefly, cells were seeded at 5 × 10^3^ cells/well in a white well plate (SPL, Korea) and then incubated at 37 °C overnight. Cells were treated with the IC_50_ of monotherapy and combination therapy for 72 h. Negative control wells were maintained untreated, and blank wells comprised media and reagents without cells. After 72 h treatment, 100 μL of caspase-Glo reagent was added to each well and mixed well (300–500 rpm, 30 s) followed by 1 h incubation in the dark at room temperature. Optimal density was measured via a luminescence microplate reader [13].

### 2.8. Statistical Analysis

All data were reported as mean ± standard deviation (SD). Statistical analysis was carried out utilizing SPSS, version 20. One-way ANOVA analysis followed by Tukey’s post-hoc test was used to compare between different treatments. A significant difference was considered when * *p* < 0.05 and ** *p* < 0.01. The combination indices (CI) of diosmetin and 5-FU on HCT116 and HT29 cells and DRI analysis were calculated using the Compusyn Software and the aforementioned equations.

## 3. Results

### 3.1. Effect of Combination on Cell Proliferation

**Figure 3:**
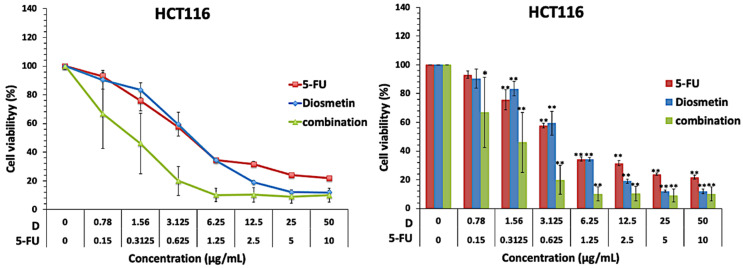
Cell growth inhibitory effect of diosmetin (D) and 5-FU as monotherapy and combination therapy in HCT116 cells. Data were expressed as mean ± standard deviation (SD). * *p* < 0.05 and ** *p* < 0.01 indicate significant differences compared to the untreated control (0).

Both monotherapy and combination therapy inhibited HCT116 cell growth in a dose-dependent manner (Figure 3). In monotherapy, the IC_50_ of diosmetin and 5-FU were 4.16 ± 1.3 and 0.83 ± 0.0 μg/mL, respectively (Table 1). Based on the IC_50_ findings of monotherapy, a fixed constant ratio of 5-FU and diosmetin (1:5) was used to perform combination therapy with a combination regimen covering the IC_50_ values, as well as higher and lower concentrations. Figure 3 shows that the combination of diosmetin and 5-FU inhibited HCT116 cell growth more potently than monotherapy, and reduced the IC_50_ of 5-FU and diosmetin to 0.27 ± 1.1 and 1.38 ± 0.8 μg/mL, respectively.

**Figure 4:**
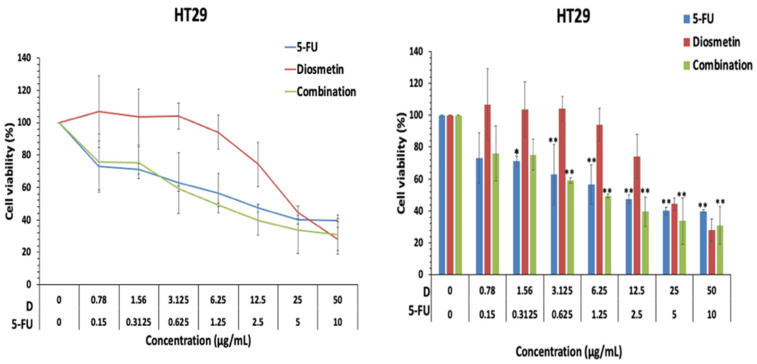
Cell growth inhibitory effect of diosmetin (D) and 5-FU as monotherapy and combination therapy in HT29 cells. Data were expressed as mean ± standard deviation (SD). * *p* < 0.05 and ** *p* < 0.01 indicate significant differences compared with the untreated control (0).

Similar to HCT116 cells, the effect of monotherapy and combination therapy was investigated in HT26 colon cancer cells. The IC_50_ of diosmetin and 5-FU as monotherapy were 22.06 ± 2.7 and 1.65 ± 0.8 μg/mL, respectively. Figure 4 shows that the combination of 5-FU and diosmetin inhibited HT29 cell growth significantly and reduced the IC_50_ of diosmetin and 5-FU to 6.03 ± 0.3 μg/mL and 1.20 ± 0.00, respectively.

### 3.2. Synergistic Effect of Combination

**Figure 5:**
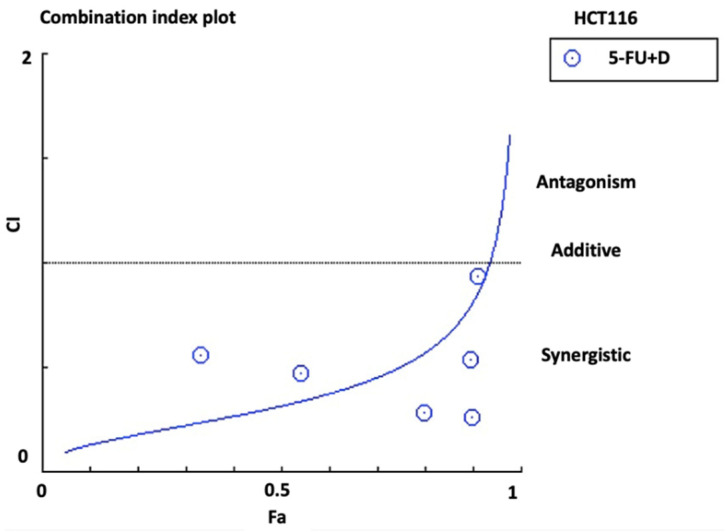
Combination index plot (Fa-CI plot) of interaction between 5-FU and diosmetin (D) in HCT116 cells generated by CompuSyn software. Fa: inhibitory effect, CI: combination index. Fa of 0.5 represents 50% growth inhibition.

The CI value for combination against HCT116 cells was calculated according to the Chou Talalay equation [15]. The mean CI value was 0.66 ± 0.4, which indicates a synergistic effect of combination therapy on HCT116 cells. Moreover, the mean DRI of 5-FU in the combination therapy was 3.0 ± 1.9, which suggests a three-fold dosage reduction compared to monotherapy. Figure 5 shows the CI plot of combination in HCT116 cells generated by CompuSyn software (version 1.0; ComboSyn, Paramus, NJ, USA), which plotted CI against the inhibitory effect. The plot revealed a synergistic pattern (CI < 1).

**Figure 6:**
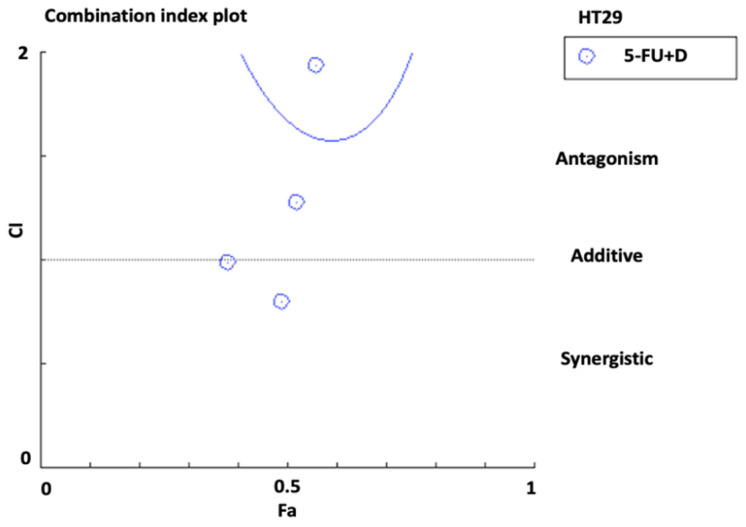
Combination index plot (Fa-CI plot) of interaction between 5-FU and diosmetin (D) in HT29 cells generated by CompuSyn software. Fa: inhibitory effect, CI: combination index. Fa of 0.5 represents 50% growth inhibition.

On the other hand, the mean CI value for combination against HT29 cells was 1.0 ± 0.2, which indicated an additive effect of combination therapy in HT29 cells. The mean DRI of 5-FU in the combination was 1.3 ± 0.5 (favorable DRI should be >1) [15]. Figure 6 shows the CI plot of combination in HT29 cells.

**Figure 7:**
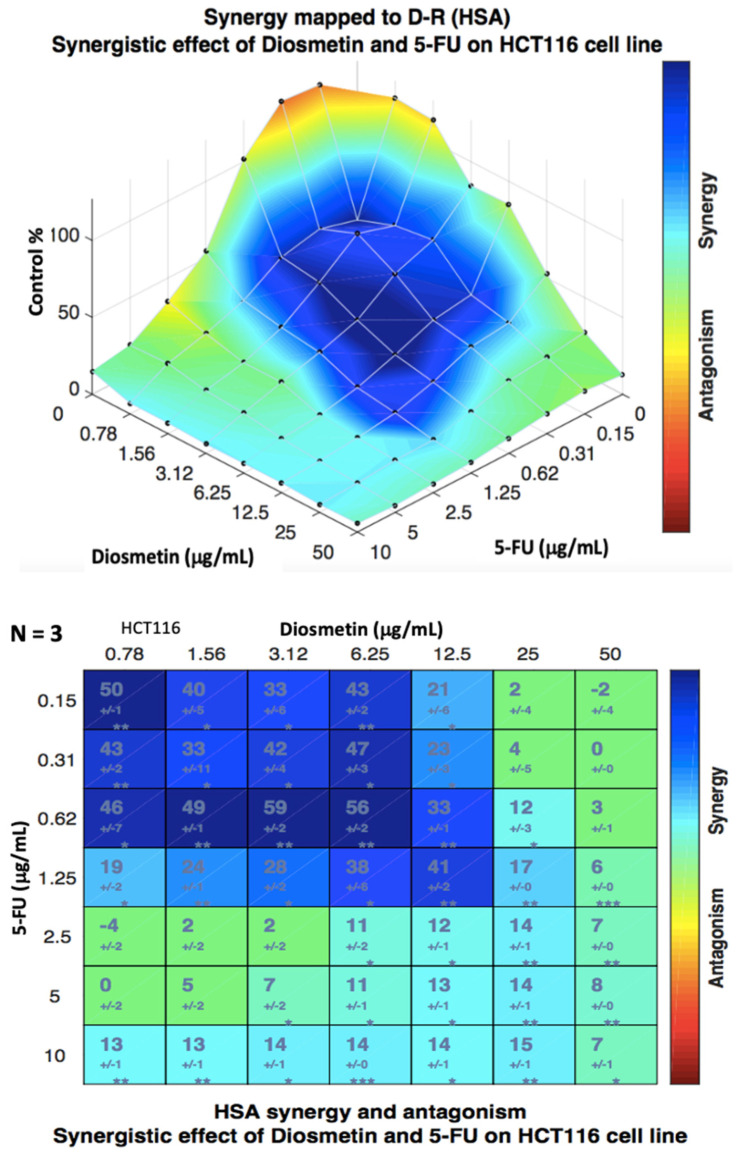
Isobologram analysis and synergism determination of combination therapy in HCT116 cells using Combenefit software. Data were obtained from three different experiments (*n* = 3).

**Figure 8:**
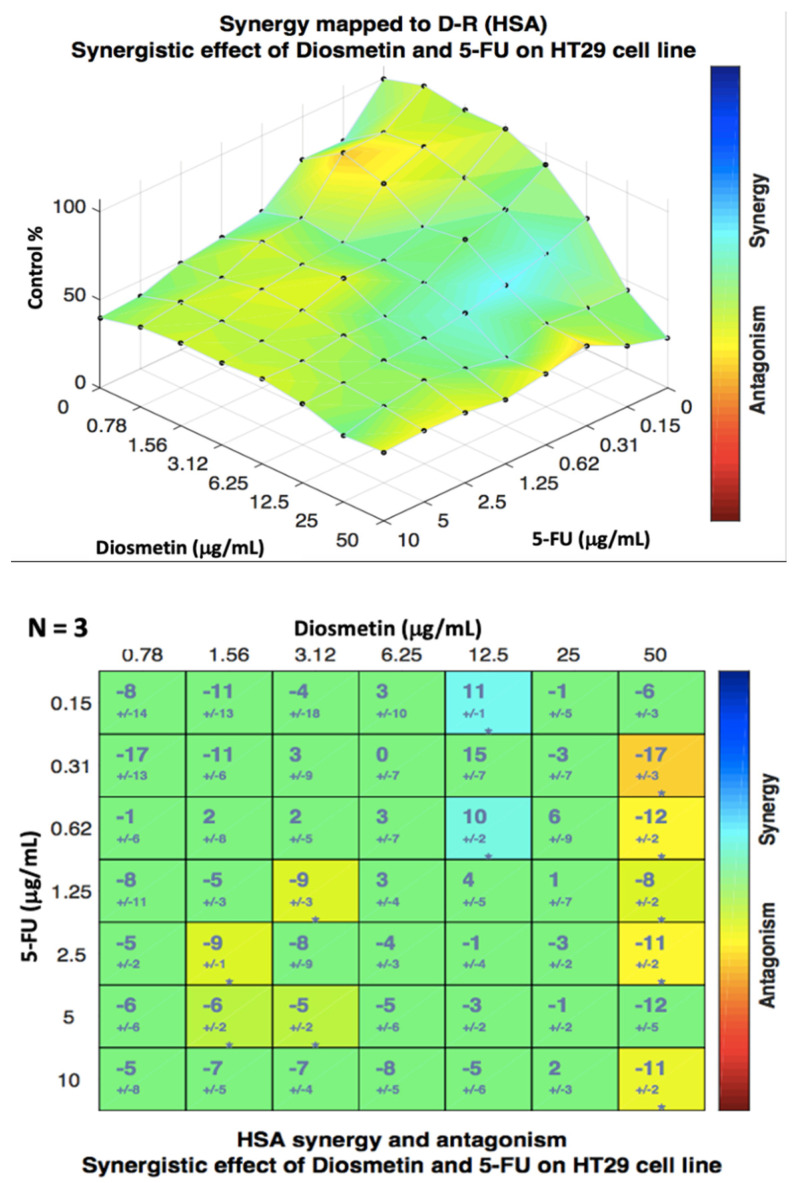
Isobologram analysis and synergism determination of combination therapy in HT29 cells using Combenefit software. Data were obtained from three different experiments (*n* = 3).

Combenefit isobologram analysis (Figure 7) confirmed the CompuSyn findings and showed a significant synergistic effect of the combination therapy in HCT116 cells at the doses between 0.15 to 1.25 μg/mL of 5-FU and 0.78 to 12.5 μg/mL of diosmetin (blue color indicates synergism) with some synergism at other doses. The same analysis was also conducted for HT29 cells (Figure 8), where the results confirmed the CompuSyn data by showing an additive effect of combined drugs at most of the doses (green color) with a low degree of synergism at the doses of 5-FU + diosmetin combination: 0.31 + 50, 0.62 + 50, 1.25 + 50, 2.5 + 50, and 10 + 50 μg/mL.

**Figure 9:**
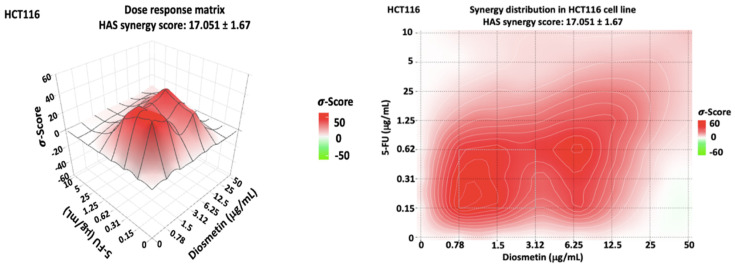
Dose–response matrix and synergy distribution in HCT116 cells treated with combination therapy. SynergyFinder 2.0 software was used for this analysis. Data were obtained from three individual experiments. The mean synergy score was 17.051 ± 1.67.

**Figure 10:**
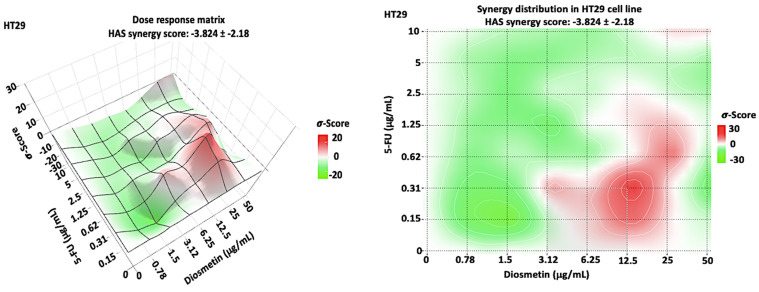
Dose–response matrix and synergy distribution in HT29 cells treated with combination therapy. SynergyFinder 2.0 software was used for this analysis. Data were obtained from three individual experiments. The mean synergy score was −3.824 ± −2.18.

Since the combenefit analysis does not provide one single mean synergy score, we used SynergyFinder 2.0 software to obtain the synergy score. The synergy score, as the average excess response to drug interactions in HCT116 cells, was 17.051 ± 1.67 (>10 indicates synergistic effect) (Figure 9). This score suggests that 17.051% of responses were beyond expectation. SynergyFinder also confirmed the synergistic doses obtained by Combenefit isobologram analysis. The same analysis was conducted for HT29 cells (Figure 10) where a synergy score was −3.824 ± −2.18 (−10 to 10 indicates an additive effect). Since this study aimed to determine the synergistic effect of the combination therapy on colon cancer cells, further investigations, as shown in the following sections, were conducted only in HCT116 cells, which showed a synergistic pattern after treatment.

### 3.3. AO/PI Double Staining Assay

**Figure 11:**
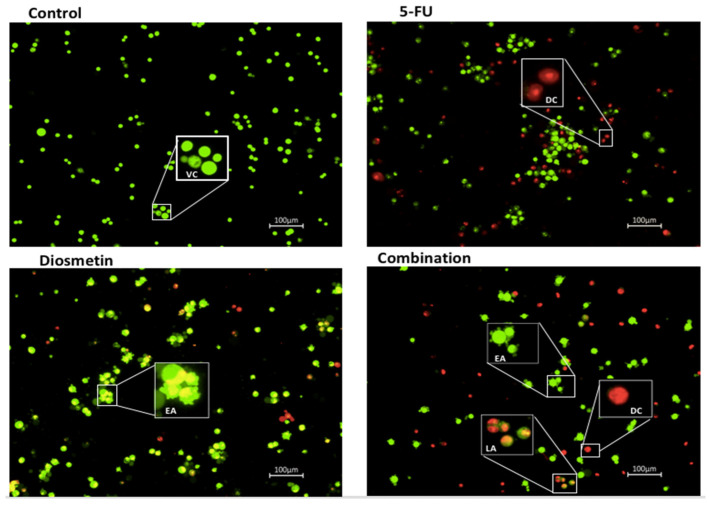
HCT116 cells stained with AO/PI after 72 h of treatment with the IC5_0_ of monotherapy and combination therapy. The control group remained untreated. VC: viable cells (green), EA: early apoptosis (green cells with blebbing), LA: late apoptosis (orange), DC: dead cells (red). 10× magnification.

The AO/PI staining findings demonstrated a difference in apoptosis induction between monotherapy, and combination therapy. HCT116 cells treated with 5-FU revealed more necrotic cells (red color). Diosmetin treatment showed signs of early apoptosis such as blebbing and chromatin condensation in HCT116 cells. Combination therapy notably resulted in both apoptotic cells and dead cells (Figure 11).

### 3.4. Annexin V-FITC Assay

**Figure 12:**
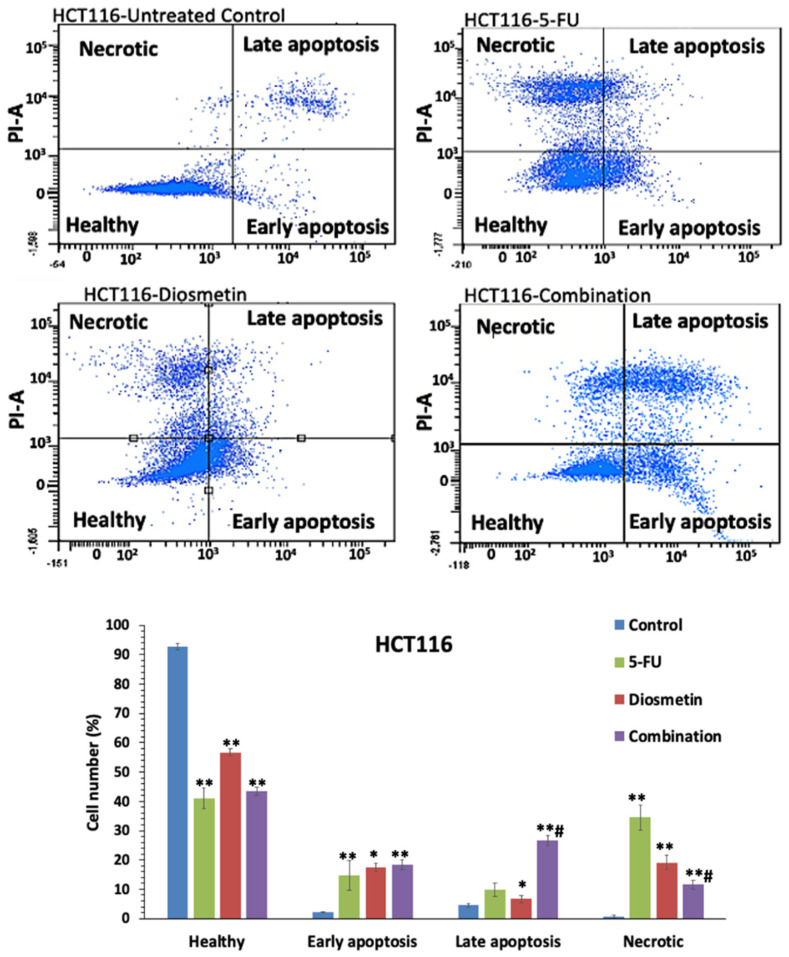
Flow cytometry graphs and apoptosis quantification in HCT116 cells after 72 h of monotherapy and combination therapy. The control group remained untreated. Total apoptotic cells (early apoptosis + late apoptosis): 5-FU: 24.6%, diosmetin: 24.1%, combination: 45%. Data are presented as mean ± SD, * *p* < 0.05, ** *p* < 0.01 indicates significant difference compared with control, # *p* < 0.01 indicates significant difference between combination-treated cells and 5-FU-treated cells.

Annexin V-FITC test was used to determine the induction of cellular apoptosis by monotherapy and combination therapy. The flow cytometry data (Figure 12) revealed that the percentage of apoptotic cells was at its highest in the combination-treated cells (45%), compared with the 5-FU treatment (24.6%) alone. 5-FU treated cells had a higher proportion of necrotic cells (34.4%), while diosmetin and combination therapy showed a lower percentage of necrosis (19.1% and 11.5%, respectively).

### 3.5. Effect of Combination on Cell Cycle of HCT116

**Figure 13:**
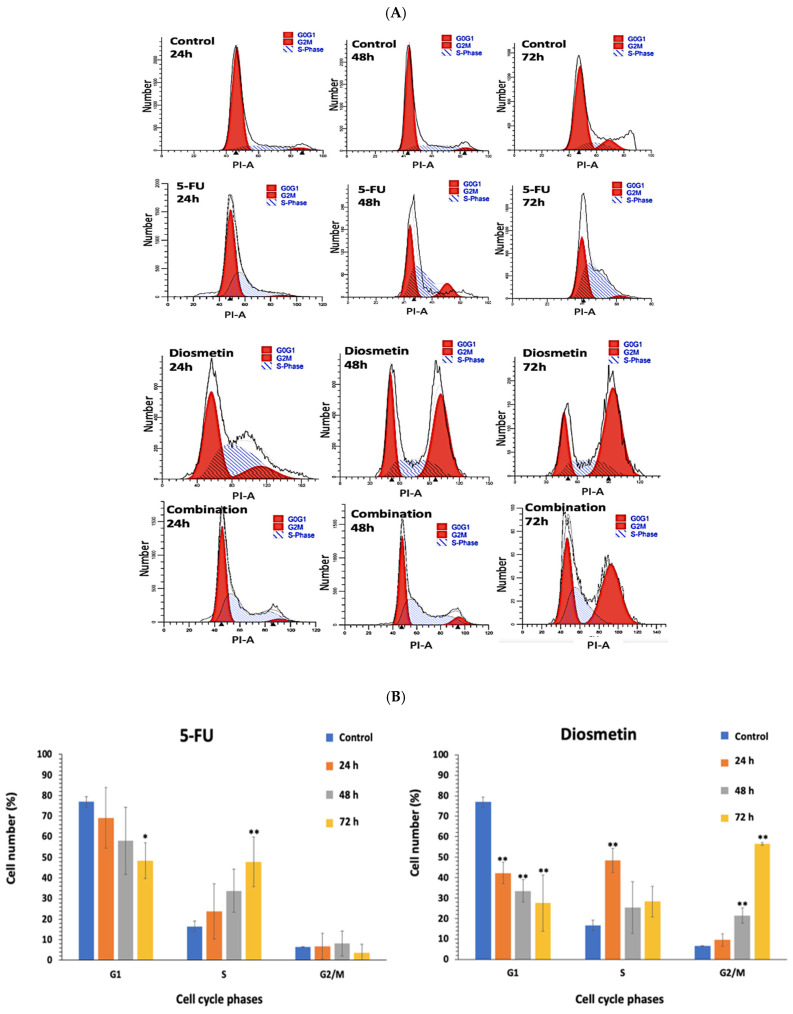

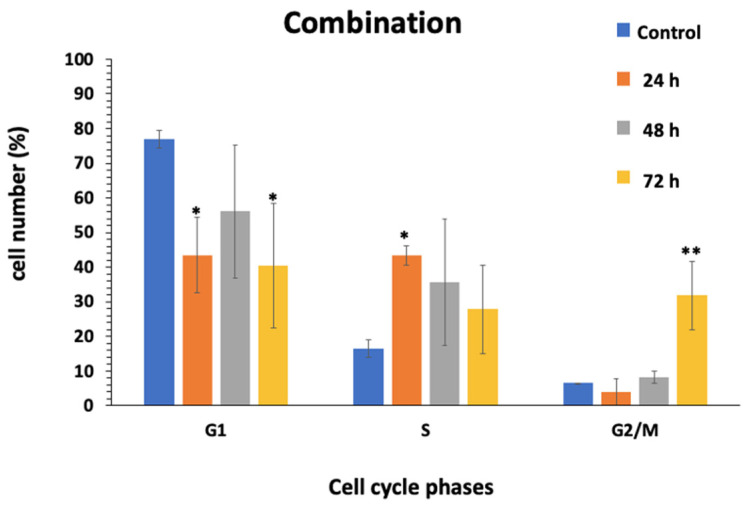
(**A**) DNA histograms of monotherapy and combination therapy on HCT116 cell cycle profile. The first and second peaks represent DNA content in G0/G1 and G2/M phases. Cell number plotted against the intensity of PI. Flow cytometric histograms are representative of 3 separate experiments. (**B**) Cell distribution percentage in each phase of HCT116 cell cycle. Data are presented as mean ± SD of 3 separate experiments, * *p* < 0.05, ** *p* < 0.01 indicate significant difference compared to control.

Flow cytometer was utilized to study the effect of monotherapy and combination therapy on HCT116 cell cycle arrest by examining the DNA content of the cells in control (untreated) and treated cells at three time points (24, 48 and 72 h). The first and second peaks in Figure 13A represent the DNA content of the cells in the G0/G1 and G2/M phases, respectively. Figure 13B compares the cell distribution percentage in each phase of the HCT116 cell cycle. Findings showed that cells treated with 5-FU alone were arrested at the S phase, and cells treated with diosmetin were arrested at the G2/M phase, while cells treated with a combination of these two drugs were arrested at the G2/M phase.

### 3.6. Effect of Combination on Caspases Pathways

**Figure 14:**
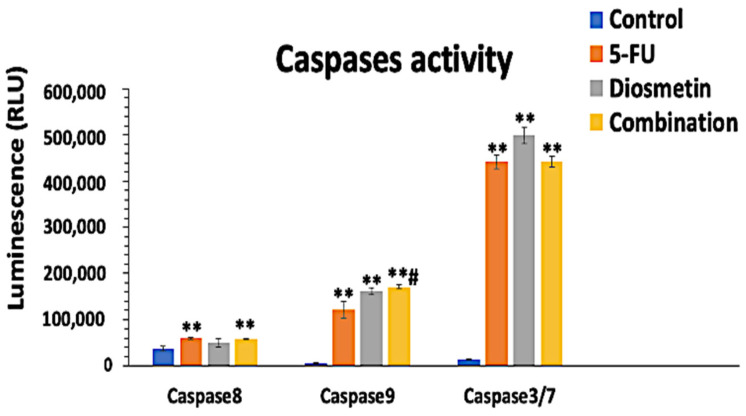
Caspase 8, 9, and 3/7 activities induced by monotherapy and combination therapy in HCT116 cells. Estimation was measured using luminescence analysis at 27 h treatment. Data are presented as mean ± SD. ** *p* < 0.01 indicates a significant difference compared with the control cells (untreated). # *p* < 0.01 indicates a significant difference between the combination-treated cells and the 5-FU-treated cells.

To further investigate the effect of monotherapy and combination therapy on apoptotic pathways, the bioluminescent intensity of caspase-8, caspase-9, and caspase 3/7 was measured after 72 h treatment. Figure 14 shows that 5-FU and combination therapy activated both caspase 8 and 9; however, the apoptotic activity of the combination was mainly through caspase 9, which suggests that the combination acts via the mitochondrial intrinsic pathway.

## 4. Discussion

Combination therapy is based on the positive effects of pharmacodynamic interactions (synergistic or additive) between two or more drugs, with synergistic interactions resulting in more effective treatments. In combination therapy, both compounds are given at lower doses and interact with multiple molecular pathways; therefore, combined treatments based on compounds that exhibit a synergistic or additive effect usually have less toxicity than monotherapy [5]. Combination therapy has shown various advantages over monotherapy, including decreasing drug concentration and toxicity, enhancing the efficacy, targeting several molecular pathways, and sensitizing cells to the treatment [6]. Therefore, in the current study, we tested the synergistic effects of diosmetin and a chemotherapeutic drug 5-FU.

Conventional chemotherapy medications are usually used in combination for the treatment of different types of cancer including CRC, this combination therapy is associated with serious adverse effects. Therefore, introducing bioactive anticancer natural compounds in combination therapy may promote chemotherapy efficacy and reduce the toxic adverse effects. In addition, natural bioactive compounds have more structural diversity, bioactivity, and complexity than synthetic drugs, and can inhibit some targets previously thought to be undruggable. They also inherently target biologically relevant pathways, because most natural bioactive compounds are secondary metabolites or signaling molecules. In addition, there is a limited overlap between the molecular signaling targeted by natural products and those targeted by synthetic drugs. This property not only indicates the potential for novel therapeutic targets for CRC but can also assist to lower the cost of developing new agents by utilizing compounds that already exist in nature and providing another option for combination therapy [16]. Furthermore, patients receiving FOLFOX regimen, which is the most regularly used chemotherapy regimen for the treatment of CRC, usually suffer from different gastrointestinal, neurological, respiratory and skin adverse effects, including hair loss [17]. Another example of conventional chemotherapy combination is the DCF (docetaxel + cisplatin + 5-FU) regimen, which is linked to stomatitis, diarrhoea, nausea, vomiting, neuropathy and associated with high toxicity [18].

The present study investigated the synergistic interaction between 5-FU and diosmetin in HCT116 and HT29 colorectal cancer cells. Since the proliferative ability of cancer cells is crucial for the tumor’s growth [19], our findings revealed that different concentrations of 5-FU and diosmetin dose-dependently inhibited the proliferation of HCT116 and HT29 cells. Moreover, based on interaction analysis using different software, combination therapy showed a synergistic effect in HCT116 cells with CI value less than one and synergy score more than 17. The IC_50_ of 5-FU reduced by 3-folds form 0.83 µg/ml to 0.27 µg/ml, which is favorable to reduce the sever toxicity and adverse effects associated with 5-FU chemotherapy. Other researchers also demonstrated that diosmetin can interact synergistically with anticancer drugs in other cancer cells, for example, diosmetin combined with paclitaxel synergistically induced apoptosis in non-small cell lung cancer cells via Nrf2 inhibition through disruption of PI3K/Akt/GSK-3β pathway [20]. Additionally, diosmetin generated a synergistic cytotoxic effect in HepG2 cells via cytochrome P450, family 1 (CYP1)-catalyzed metabolism, activation of c-Jun N-terminal kinase (JNK)/extracellular signal-regulated kinase (ERK), and *p53/p21* overexpression [21]. On the other hand, in our study the combination therapy induced an additive effect in HT29 cells with CI value equal to one and synergy score of −3.824. Although both cell lines are colorectal cancer cells, HT 29 and HCT-116 represent different extents of mutation and differentiation. HT29 is a p53 mutated type and has an intermediate capacity to differentiate into enterocytes and mucin-expressing lineages, while HCT116 is known to be a highly aggressive wild type cell line that shows no ability to differentiate. Different interactions effects of 5-FU and diosmetin combination on these two cancer cell lines could be attributed to the differences in mutation and differentiation, however the additive effect in HT29 cells still contributes to the beneficial effects of this combination. Another possible reason for the different interaction effect in HCT116 and HT29 cells could be due to the differences in their genetic profiles. Sensitive p53 wild type cancer cells such as HCT116 are usually targeted via p53-mediated apoptosis while mutated or null p53 cells such as HT29 cells can be inhibited by drugs that induce p53-independent cell death pathways [22]. Further molecular mechanism involved in the effect of the combination treatment on these two cell lines are warranted to understand such activities. 

The HAS synergistic model was selected in both Combenefit and SynergyFinder analysis of the combination therapy as this model definition matches the design of this study. This synergistic model assumes that the expected effect of the combination therapy is equivalent to the higher individual drug effect at the dose in the combination. This represents the idea that a synergistic drug combination produces additional effects compared to what its components can achieve alone [23]. 

Imbalance between proliferation and apoptosis is a major element in the initiation of cancer. Apoptosis serves as a key function in correcting normal tissue stability [24]. Therefore, therapy options that target apoptosis may be effective in preventing the progression of CRC. Apoptosis is characterized by cell shrinkage, chromatin, nuclear condensation, and plasma membrane blebbing [25]. AO/PI staining and Annexin V-FITC were conducted to detect whether the suppression of HCT116 cells proliferation induced by combination therapy was associated with apoptosis. In our study, the treated cells exhibited apoptotic characteristics, including membrane blebbing in early apoptosis and chromatin condensation in late apoptosis. Before the cellular membrane disintegrates during early apoptosis, phospholipid asymmetry occurs [26,27]. Phosphatidylserine (PS) translocates to the outer plasma membrane, where it is exposed to the exterior surface. As a result, PS translocation can be used to investigate apoptosis. Annexin V is a calcium-dependent phospholipid-binding protein with a high affinity for PS, and it is frequently used in conjunction with PI (fluorescent dye) to identify apoptotic and necrotic cells [19]. To further quantify the apoptotic HCT116 cells following the treatment with monotherapy and combination, cells were exposed to Annexin V/PI staining and subjected to flow cytometry. Combination therapy significantly increased apoptotic cells to 45%, compared with 5-FU-treated cells, which showed only 24.6% of apoptosis. There were more necrotic cells (34.4%) in HCT116 cells treated with 5-FU than combination-treated cells (19.1%). It has been reported that chemotherapeutic drugs not only trigger apoptosis, but also other types of cell suicide, such as necrosis, which triggers further inflammation. Thus, it is not a preferred pathway for cancer treatment [28]. Therefore, the combination of 5-FU and diosmetin has the advantage to act through activating the apoptosis pathway with less impact on the necrosis pathway compared with 5-FU.

Several dysregulated signaling pathways have been linked to cancer development. Conventional chemotherapy agents have toxicity and severe adverse effects. Therefore, finding new multi-targeted treatment to reduce cancer’s dysregulated signaling is critical [29]. Diosmetin was reported to have a potential effect on signaling pathways involved in colorectal cancer. These pathways include apoptosis, TGF-β/BMP, NF-_k_B [13], PI3K/AKT [30], and Notch signaling pathways [31]. In our study, a combination of diosmetin with chemotherapeutic drug (5-FU) could target multiple signaling pathways and produce a higher response rate against colorectal cancer. Combination therapy has shown to be significantly effective in terms of anti-cancer management. Its superiority arises from its capacity to target many pathways, and reducing drug resistance to a minimum. Pathway dysregulation in cancer cells, as well as the alteration of homeostatic settings, all contribute to the unregulated proliferation. For example, mutations in tumor suppressor genes such as p53, which normally activates cell cycle arrest when DNA is damaged, resulting in the accumulation of damaged DNA and the inhibition of cell cycle arrest, contribute to an increase in the rate of cell proliferation. Additionally, in cancer cells, upregulated autocrine growth factor production or an upregulated autocrine loop can contribute to tumor cell growth [32]. In colon cancer cells, a similar effect can be seen [33]. When it comes to autocrine growth factors, if VEGF is upregulated it can lead to metastasis, which can make the prognosis for survival worse [32,34]. Therefore, targeting several pathways with a multiple-agent combination can enhance the treatment while lowering the risk of cancer cells becoming more aggressive and incurable. In addition, the doses of each drug/compound in combination therapy can be lowered, resulting in fewer adverse effects compared with monotherapy [35]. Another benefit of combination therapy is that different drugs can target the heterogeneous character of tumors, boosting the chances of killing cancer cells, including the cancer stem cell population, which has been linked to drug resistance and cancer recurrence following remission [36,37,38]. 

Cell cycle arrest of cancer cells at different phases can inhibit its proliferation, and may lead to cell death; thus, describing the profile of mitotic disruption and cells’ arrest is critical for a complete and reliable characterization of the interaction mechanism of combination [5]. The cell cycle in cancer cells is linked to significantly altered genes, which can lead to an excessive proliferation. As a result, therapies that target cell cycle components are one of the cancer-fighting strategies. Anticancer drugs should therefore target proteins that disrupt the mitotic spindle function. Anticancer medications that modify mitotic components cause mitosis slippage and arrest cancer cells in the G2/M phase [13]. Many researchers have highlighted the impact of cell cycle blockage on the induction of the synergistic cytotoxic effect. It was suggest that the arrest of the cell cycle phase, typical for an anticancer drug, is necessary for the induction of a synergistic interaction between anticancer drugs and natural compounds such as resveratrol combined with 5-FU against HCT116 colon cancer cells [5]. This was also demonstrated in a study in HT29 cells after treatment with sulforaphane and 5-FU [39]. The G1 checkpoint is often used by normal cells to repair DNA damage. Tumor cells, on the other hand, rely on the G2 checkpoint to defend themselves from DNA damage [40]. This highlights the G2 checkpoint as a specific target for cancer treatment. A highly conserved protein kinase family controls the cell cycle. Cyclins activate CDKs by building complexes with them, the most essential of which being the cyclin B1/CDK1 complex for the G2 to M phase transition. Reduced production of the cyclin B/CDK1 complex during cell cycle progression is known to cause G2/M phase arrest [40]. The G2 checkpoint is important for suppressing cancer progression because it prevents cells from entering mitosis when DNA is damaged, providing an opportunity for repair or stopping the proliferation of damaged cells. This checkpoint is crucial in understanding the molecular etiology of cancer, since it helps to maintain genomic integrity [41,42]. In our study, the flow cytometry method was further applied to investigate the cell distribution percentage in each phase of the HCT116 cell cycle after monotherapy and combination therapy. As a result, combination therapy increased the proportion of cells in the G2/M phase over time. However, whether combination therapy inhibited G2/M transition or induced M phase arrest needs to be studied further by investigating the expression level of phospho-CDK1, and the B/CDK1 complex.

The initiation of the caspase cascade is essential for the induction of apoptosis [19]. Activation of apoptosis depends on the involvement of at least two distinct pathways; extrinsic and intrinsic mitochondrial apoptotic pathways, which are associated with the activation of caspase 8 [43] and caspase 9 [19], respectively. Apoptosis can be activated in response to a variety of death cues generated from within the cells, including oncogene activation and DNA damage. The intrinsic route is mediated by mitochondria, and many proteins are released from the intermembrane space of mitochondria into the cytosol in response to apoptotic stimuli. Cytochrome C, SMAC/DIABLO, AIF, EndoG, and Bcl-2-family proteins are among the well-studied proteins. Cytochrome C interacts with the protein APAF1 and causes a conformational change, allowing APAF1 to bind to ATP/dATP and form the apoptosome, which activates caspase-9, and other caspase cascades. Although the majority of chemotherapies activate intrinsic apoptosis, the interaction between the extrinsic and intrinsic apoptosis pathways might result in a synergic and efficient cell death induction. In numerous cancer cells, this synergy between the death receptor and the mitochondrial pathway has been observed [44]. 

The activation of caspases-8, 9, and 3/7 in HCT116 cells were explored in this work to determine which apoptotic pathway is triggered in cells treated with monotherapy and combination therapy. The apoptotic effect of the combination therapy was elicited by caspase-8 and 9 activations, followed by caspase 3/7 activation, which indicates the involvement of both extrinsic and intrinsic apoptosis pathways. This suggests that combination therapy could be a better treatment option, since it requires lower doses of 5-FU to achieve the same therapeutic effect as monotherapy, hence reducing 5-FU toxicity. This study also revealed that combination therapy induces apoptosis through different apoptosis pathways. However, more research at the protein expression level is needed to demonstrate the multiple-pathway targets of combination therapy underlying these activities.

## 5. Conclusions

In conclusion, diosmetin in combination with 5-FU has a synergistic effect in HCT116 colon cancer cells and an additive effect in HT29 cells. Combination therapy enhances the efficacy of 5-FU and reduces its unfavorable adverse effects, which was demonstrated by high DRI and a high synergy score in HCT116 cells. The combination of 5-FU and diosemetin activates apoptosis mainly via the intrinsic pathway and arrests HCT116 cells at the G2/M phase. Further studies are required to assess the underlying mechanism of action of the combination, and to confirm the anti-tumorigenic activities of this combination in an appropriate animal model.

## Figures and Tables

**Table 1:** IC_50_ of diosmetin and 5-FU as monotherapy or combination therapy in HCT116 and HT29 cells.

Treatment	Drug	HCT116 Cells IC_50_ (μg/mL)	HT29 Cells IC_50_ (μg/mL)
Monotherapy	Diosmetin	4.16 ± 1.3	22.06 ± 2.7
	5-FU	0.83 ± 0.0	1.65 ± 0.8
Combination therapy	Diosmetin	1.38 ± 0.8	6.03 ± 0.3
	5-FU	0.27 ± 1.1	1.20 ± 0.0

## Data Availability

Not applicable.
